# Potential Impact of Flow Cytometry Antimicrobial Susceptibility Testing on the Clinical Management of Gram-Negative Bacteremia Using the FASTinov^®^ Kit

**DOI:** 10.3389/fmicb.2017.02455

**Published:** 2017-12-12

**Authors:** Sofia Costa-de-Oliveira, Rita Teixeira-Santos, Ana P. Silva, Elika Pinho, Paulo Mergulhão, Ana Silva-Dias, Nádia Marques, Inês Martins-Oliveira, Acácio G. Rodrigues, José A. Paiva, Rafael Cantón, Cidália Pina-Vaz

**Affiliations:** ^1^Division of Microbiology, Department of Pathology, Faculty of Medicine, University of Porto, Porto, Portugal; ^2^CINTESIS - Center for Health Technology and Services Research, Faculty of Medicine, University of Porto, Porto, Portugal; ^3^FASTinov, S.A., Matosinhos, Portugal; ^4^Department of Emergency and Intensive Care, Centro Hospitalar Sao Joao, Porto, Portugal; ^5^Department of Medicine, Faculty of Medicine, University of Porto, Porto, Portugal; ^6^Servicio de Microbiología, Hospital Universitario Ramón y Cajal and Instituto Ramón y Cajal de Investigación Sanitaria, Madrid, Spain

**Keywords:** flow cytometry, antibacterial susceptibility, de-escalation, bacteremia, blood culture

## Abstract

Laboratory assessment of antimicrobial susceptibility is a prerequisite for adequate management of infections. The aim of this research was to evaluate the performance of the novel FASTinov^®^ kit for antimicrobial susceptibility testing (AST) of Gram negative bacilli directly on positive blood cultures. One hundred and two positive blood cultures from patients of a Portuguese University Hospital were included. AST were performed with routine method, Vitek2, with FASTinov^®^ kit, and with the gold standard microdilution. Bacteria directly extracted from blood cultures were used to inoculate the FASTinov^®^ kit. Time-to-result as well as the number of patients receiving initially inappropriate therapy (and those in whom de-escalation would have been done) and length of stay (LOS) was recorded. Seventy percent of patients were over 70 years old and 18.6% were admitted in intensive care units. Regarding the isolates, 88.2% were Enterobacteriaceae, 9.8% *Pseudomonas* spp. and 1% *Acinetobacter* spp. Extended spectrum β-lactamases producing-Enterobacteriaceae were found in 7.8% of cases and 10.8% were multi-drug resistant. Fifty-one hours was the mean of time-to-result for routine test (Vitek2) vs. 2 h response regarding Fastinov^®^ test. The overall agreement between FASTinov^®^ and the reference microdilution method was 98%. According to the susceptibility phenotype, 16.7% of patients received initially inappropriate therapy and the mean hospital LOS of these patients was significantly higher. FASTinov^®^ kit revealed an excellent correlation with the AST standard method and provided much earlier results than Vitek2.

## Introduction

Bloodstream infections (BSI) are a serious clinical adverse events associated with very high morbidity and mortality. They represent one of the most common health care related infections and rank in the top four most costly conditions. The diagnosis of BSI is based upon blood cultures, with automated detection of the presence of viable microorganisms. Matrix-assisted laser desorption/ionization time-of-flight (MALDI-TOF) mass spectrometry can allow quick identification of the responsible organism after its culture (within 24 h after the positive flag) (Bizzini et al., 2010; Greub, 2010) but it could also be performed directly from positive blood cultures (Prod'hom et al., 2010; Clerc et al., 2013), thus saving considerable time. Recently, Marschal et al. have described the Accelerate Pheno system as an automated test system capable of performing both identification and AST directly from positive blood cultures of Gram-negative organisms within approximately 7 h (Marschal et al., 2017). Near-future alternatives of antimicrobial susceptibility tests with answer less than 5 h include MALDI-TOF, due to its ability to detect some mechanisms of resistance (Burckhardt and Zimmermann, 2011), and flow cytometry (Alvarez-Barrientos et al., 2000; Pina-Vaz and Rodrigues, 2010). Molecular methods can also contribute to a rapid and specific identification of microorganisms. However, concerning susceptibility testing, scarce useful information can be provided since only a few genes codifying for resistance-associated proteins are yet known (Salimnia et al., 2014; Ceyssens et al., 2016). A global susceptibility profile is impossible to be obtained by molecular biology unless whole genome sequencing tools are used (Zankari et al., 2013).

The emergence and spread of antimicrobial resistance constitutes a major medical problem. Laboratory assessment of antimicrobial susceptibility is a prerequisite for adequate management of infections. However, prolonged time is required for routine phenotypic antimicrobial susceptibility testing (AST) since growth in the presence of different antimicrobial drugs is mandatory. Flow cytometry (FC) represents an efficient and fast approach for the analysis of cell architecture and functional phenotypes, with considerable advantages over conventional methods. By studying structural lesions induced by drugs that lead to changes in morpho-functional parameters (e.g., membrane potential, cell size, amount of DNA), antimicrobial susceptibility can be assessed without the need of prolonged microbial growth. The availability of such an assay for susceptibility testing in critical bloodstream infections is hereby proposed, since it can be performed in just a few hours, with high accuracy. We previously described microbiological applications of FC, including susceptibility evaluation of antifungals, as well as the elucidation of antifungal resistance mechanisms (Pina-Vaz and Rodrigues, 2010). Moreover, we developed AST cytometric protocols regarding the main mechanisms of resistance to betalactams with the most important bacteria involved in clinical infections (Faria-Ramos et al., 2013; Silva et al., 2016). In this study we evaluate the performance of FASTinov^®^ kit for AST of Gram negative bacteria from positive blood cultures. In collaboration with members of the antimicrobial stewardship program of our university hospital, we also determined if the anticipation of AST results (less 46 h) would have led to changes in empiric antibiotic therapy and, consequently, de-escalation of treatment.

## Materials and methods

### Study design and sample collection

**Figure 1:**
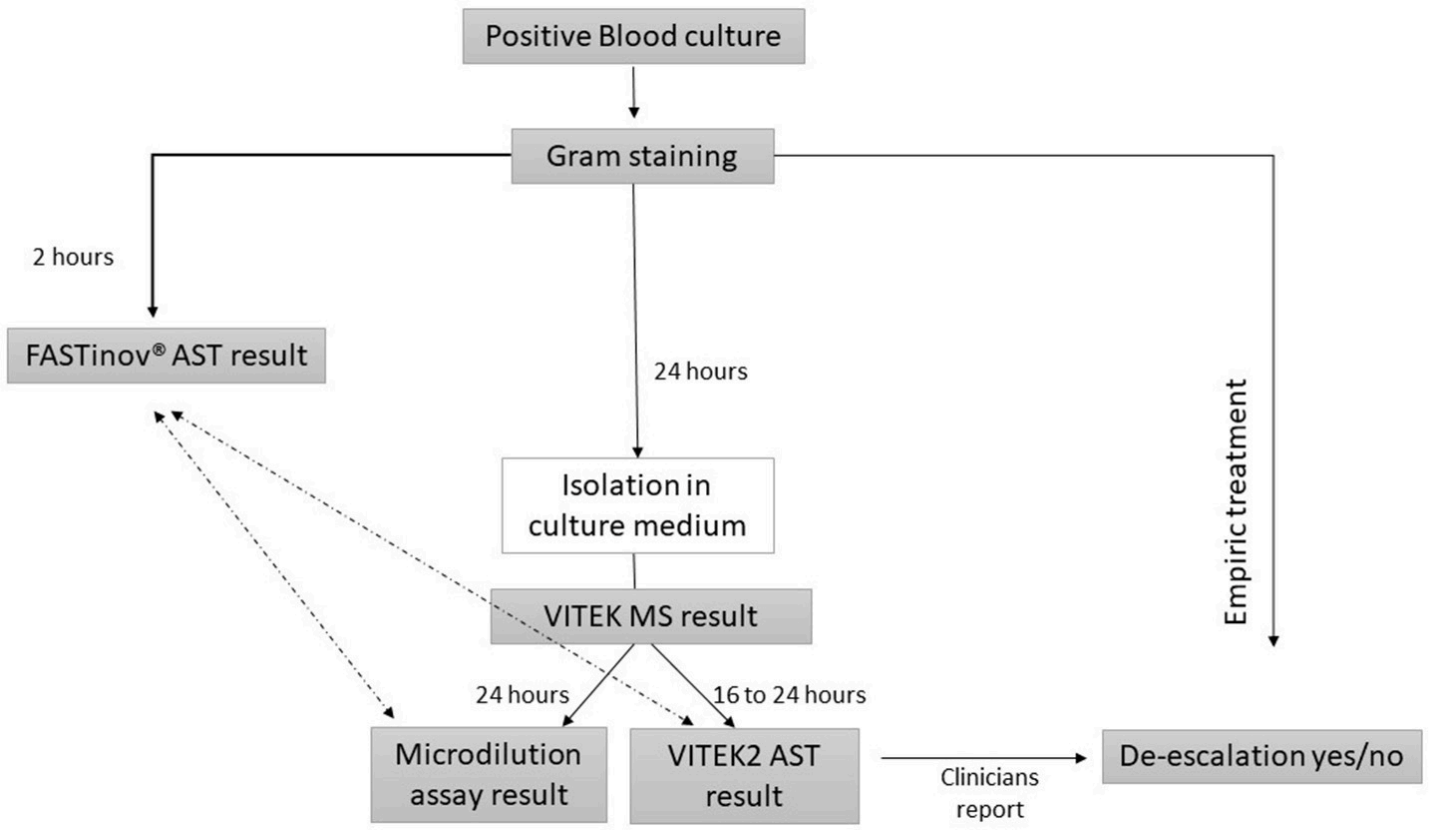
Flowchart representing the overall study design. Only VITEK2 AST (BioMérieux) result was provided to clinicians. The stewardship team involved in the study evaluated the potential impact of FASTinov^®^ kit (around 23 h earlier) on the clinical management of Gram-negative bacteremia. The FASTinov^®^ AST result was compared with VITEK2 and the gold standard microdilution assay (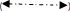).

The present study was conducted from March to July 2015 at Centro Hospitalar S. João in Porto, Portugal (a 1000-bed University hospital). Positive blood culture bottles were managed by BACTEC 9240 automated blood culture system (Becton Dickinson) which detects bacterial growth by fluorescent sensor technology. The content of each bottle flagged positive by the BACTEC instrument was initially Gram stained and cultured according the routine protocol; in parallel FASTinov^®^ kit for gram negative bacteria (developed by FASTinov S.A.) was performed directly from positive blood cultures. The general scheme of the study design is represented in Figure 1. A single positive blood culture bottle from each patient was studied. Bottles with mixed Gram-staining morphologies were excluded. A total of 102 consecutive positive blood culture sets was included during the study period. Clinical data such as age, sex, care unit of admission, length of stay, initial empiric and subsequent antimicrobial prescription and clinical outcome was registered (Table 1).

**Table 1:** Data from 102 patients yielding positive blood cultures (March-July, 2015) included in the study and receiving empiric antibiotic therapy.

**Variables**	***N* (%)**	**Appropriate therapy**	**Inapropriate therapy^a^**
Patients	102 (100)	85 (83.3)	17 (16.7)
Male gender	55 (53.9)		
Age, mean of years (range)	65.8 (19–92)	64.5 (19–92)	72.5 (43–87)
**HOSPITAL DEPARTMENT OF ADMISSION AT TIME OF INFECTION**			
Medicine	14 (13.7)	9 (10.6)	5 (29.4)
Surgery	9 (8.9)	6 (7.0)	3 (17.7)
Intensive/intermediate care unit	19 (18.6)	19 (22.4)	–
Emergency department	56 (54.9)	47 (55.3)	9 (52.9)
Other	4 (3.9)	4 (4.7)	–
Hospital length of stay, median of days (range)	11 (0–165)	10 (0–165)	26 (0–111)
**ANTIMICROBIAL THERAPY**			
Single drug	72 (70.6)	58 (68.2)	14 (82.4)
2 drugs	27 (26.5)	24 (28.2)	3 (17.6)
More than 2 drugs	3 (2.9)	3 (3.5)	–
**CLINICAL OUTCOME**			
Death	14 (13.7)	10 (11.8)	4 (23.5)

a *Inappropriate therapy was considered when a drug for which the bacteria was resistant was used as empirical antibiotic treatment (Vallés et al., 2003)*.

### Control resistant strains

Since resistant strains (required to detect very major errors) could be insufficiently represented in clinical samples, additional well-characterized control strains (*n* = 27) were also considered for testing in parallel (Table 2). For this purpose, aerobic blood culture bottles were spiked with 2 × 10^3^ cells/bottle and fresh blood (Puttaswamy et al., 2011). After the Bactec incubation, such positive bottles were processed as the routine protocol and FASTinov^®^ kit recommendations.

**Table 2:** Gram negative bacilli recovered from positive blood cultures and resistance pattern according to standard reference microdilution protocol and EUCAST guidelines interpretation.

	**Clinical isolates (*N*)**	**Control strains (*N*)**	**Total (*N*)**
ESBL-positive	8	12	20
Carbapenemase-positive	–	11	11
AmpC-positive	–	4	4
**RESITANCE PATTERN**			
Amikacin	4	12	16
Amoxicillin + clavulanic acid	23	27	50
Cefotaxime	11	27	38
Ceftazidime	10	27	37
Ciprofloxacin	22	23	45
Colistin	3	0	3
Gentamicin	18	18	36
Meropenem	1	15	16
Piperacillin + tazobactam	15	27	42

### Species identification

Identification of all clinical isolates included in this study was performed, after sufficient growth on agar plates, by mass spectrometry (Vitek MS, bioMérieux) in accordance to the manufacturer's instructions. The results were available 24 h after the blood culture bottle was flagged positive.

### Routine antimicrobial susceptibility testing

Routine AST was performed from overnight subcultures using Vitek2, with different cards according to Vitek MS result, namely AST-192 (for Enterobacteriacae and *Acinetobacter* spp.) and AST-222 (for *Pseudomonas* spp.). Whenever technical problems occurred with Vitek2 results, additional tests such as disk diffusion test or Etest were performed, requiring further 24 h.

### FASTinov^®^ kit for gram negative

The FASTinov^®^ gramneg kit was used after Gram stain observation (immediately after blood culture was flagged positive) and before definitive identification by Vitek MS (see Figure 1). The FASTinov^®^ gramneg kit includes a microplate containing a panel of lyophilized antimicrobials, a specific fluorochrome and dedicated software (US Patent n° 9,290,790 B2). It was inoculated according to manufacturer instructions and incubated for 1 h at 37°C with shaking. Afterwards, flow cytometric analysis was performed on a BD Accuri^TM^ C6 (BD Biosciences) flow cytometer and the AST result was automatically categorized as susceptible (S), intermediate (I) or resistant (R) (see Figure S1). The dedicated software allows the selection of both EUCAST and CLSI classification rules. For the purpose of this study we used EUCAST recommendations (EUCAST, 2015) since such rules are followed by the routine hospital laboratory using Vitek2 system.

### Broth microdilution assay

Following the selection of the antimicrobial panel available in both automated methods (Vitek2 and FASTinov^®^), microdilution assay was performed according to ISO recommendations and considered the gold standard method.

### Comparison between methods

All isolates tested were grouped into the categories S, I or R according to EUCAST AST breakpoints (EUCAST, 2015). Categorical analysis was performed between the ISO method and FASTinov^®^ or Vitek2. Agreement of susceptible, intermediate and resistant results between a breakpoint test in both methods and the reference method was calculated and errors classified as very major errors (VMEs; a false susceptible), major errors (ME; a false resistant) and minor errors (mEs; any false result involving an intermediate result).

### Time-to-result

The time from blood culture positivity until the availability of AST results by FASTinov^®^ kit and Vitek2 method was recorded.

### Impact of Fastinov^®^ result

A trained stewardship team was asked to compare the empirical therapy prescribed to patient (based upon Gram staining) with the most adequate therapy according to the susceptibility phenotype profile given by the FASTinov^®^ kit, which results were obtained 48 h before Vitek2 results. The percentage of cases of inadequate therapy was calculated, as well as the percentage of patients in whom de-escalation would have been possible according to AST results.

### Ethical approval

This work was approved by the Ethical Commission of Centro Hospitalar São João (CE 27-15), where it was stated that the study subjects were anonymized and no written informed consent was needed.

### Statistical analysis

In order to evaluate the agreement between FASTinov^®^ kit and Vitek2 vs. reference method, the categorical agreement (CA) was calculated. The percentage of FASTinov^®^ gramneg kit error was determined using the clinical and control strains with confidence interval at 95%. Very major error rates were calculated using the total number of resistant strains as the denominator, whereas major error (ME) rates were calculated using the total number of susceptible strains as the denominator (Jorgensen, 1993; CLSI, 2016). The comparison between hospital length of stay (LOS) and appropriate/inappropriate therapy was evaluated with Kruskal-Wallis test. For comparison between the outcome and appropriate/inappropriate therapy, the Chi-square test was used. A *p*-value inferior to 0.05 was considered as statistically significant.

For calculation of all measures, including the percentage error and descriptive statistics, the SPSS program (version 23.0) was used.

## Results

Table 1 aggregates data from patients included in the study regarding, age, gender, hospital department of admission, length of staying (LOS), empiric antimicrobial therapy and clinical outcome. Almost 17% of the patients received inappropriate therapy, which was associated with a 16-day increase of hospital LOS. In 10 patients receiving initially inadequate therapy and in whom antibiotics were changed, such change occurred, on average 2.8 days after the initial prescription and was made either before the routine AST result was available (suggesting that this decision was based on risk factors and clinical evolution) or (5/10 cases) on the same day that AST results became available. In this last instance, patients received inappropriate therapy during 4.2 days, on average. De-escalation was deemed possible in 65 out of 85 (76.5%) patients, but was done in only 23 (27.1%) of them. De-escalation occurred, on average, 4.5 days (0–10) after the start of antibiotic therapy and in most cases (20/23) only after AST result was known. Regarding the outcome, data analysis revealed no significant differences among patients who had received appropriate/ inappropriate therapy (*p* = 0.455).

The drug most frequently used was piperacillin-tazobactam (27.4%) followed by ceftriaxone (24.5%); when combination therapy was used, piperacillin-tazobactam plus amikacin (4.9%), imipenem plus vancomycin (4.9%) and piperacillin-tazobactam associated with vancomycin and amikacin (2%) were the most frequent drug combinations.

Regarding AST time-to-result, the routine laboratory method (Vitek2) required on average 51 h (48–58 h). This range of time was often associated with the need to perform additional susceptibility testing, such as agar disk diffusion or Etest in several clinical isolates to confirm results. FASTinov^®^ results were available within a range of 2 h.

Table 2 details the Gram-negative bacilli studied (isolated from blood cultures and those spiked with selected control strains), the respective resistance phenotype, the number of ESBL, carbapenemases and AmpC positive strains, and the number of multiresistant strains based on ISO microdilution methods. Regarding the clinical isolates, 88.2% were Enterobacteriaceae (60% *E. coli*, 18.9% *K. pneumoniae*, 3% *Enterobacter* spp., 1% *Citrobacter* spp 2% *Proteus* spp. and 3% *Serratia* spp). Regarding non-Enterobacteriaceae, *Pseudomonas spp* were the most isolated species followed by *Acinetobacter spp* (9.8 and 1%, respectively).

The FASTinov^®^ gramneg kit results are detailed in Table 3. The highest CA was observed for colistin (1.00), followed by piperacillin/tazobactam, gentamicin, meropenem and cefotaxime (0.98). A low CA was detected for ciprofloxacin (0.94), with a major error rate of 3.57%. A high major error rate was also observed for amoxicillin-clavulanic acid (3.80%). The very major discrepancies were verified for meropenem (6.25%), gentamicin (2.78%), and amoxicillin-clavulanic acid (2.00%). Regarding ESBL detection, the CA between FASTinov^®^ gramneg kit and ISO microdilution was 1.00.

**Table 3:** Categorical agreement between FASTinov^®^ Gram negative test or Vitek2 (bioMérieux) and broth microdilution; FASTinov ^®^ gramneg kit error rate.

**Agreement with microdilution**	**FASTinov^®^**	**Vitek2**^a^	**No. (%) mE**	**95% CI for mE (%)**	**No. (%) ME**	**95% CI for ME (%)**	**No. (%) VME**	**95% CI for VME (%)**	**Broth microdilution AST**	
									**Susceptible**	**Resistant**
Amikacin	0.97	0.93	2 (1.55)	(0.19: 5.49)	2 (1.77)	(0.21: 6.25)	–	–	113	16
Amoxicillin+clavulanic acid	0.97	0.94	–	–	3 (3.80)	(0.79: 10.70)	1 (2.00)	(0.05: 10.65)	79	50
Cefotaxime	0.98	1.00	1 (0.78)	(0.02: 4.24)	1 (1.10)	(0.03: 5.97)	–	–	91	38
Ceftazidime	0.97	0.94	2 (1.55)	(0.19: 5.49)	2 (2.17)	(0.26: 7.63)	–	–	92	37
Ciprofloxacin	0.94	0.96	5 (3.88)	(1.27: 8.81)	3 (3.57)	(0.74: 10.08)	–	–	84	45
Colistin	1.00	1.00	–	–	–	–	–	–	126	3
Gentamicin	0.98	0.96	–	–	1 (1.08)	(0.03: 5.85)	1 (2.78)	(0.07: 14.53)	93	36
Meropenem	0.98	1.00	1 (0.78)	(0.02: 4.24)	1 (0.88)	(0.02: 4.83)	1 (6.25)	(0.16: 30.23)	113	16
Piperacillin+tazobactam	0.98	0.95	1 (0.78)	(0.02: 4.24)	1 (1.15)	(0.03: 6.24)	–	–	87	42
Overall	0.98	0.97	12 (1.03)	(0.54: 1.80)	14 (1.59)	(0.87: 2.66)	3 (1.06)	(0.22: 3.07)	878	283
ESBL I detection	1.00	1.00								

a *Result obtained from colonies*.

*mE, minor errors; ME, major errors; VME, very major errors;–, no error; CI, confidence interval*.

We found a higher agreement between the gold standard and FASTinov^®^ kit (0.98) than between the gold standard and Vitek2 (0.97).

## Discussion

Our study clearly evaluates the performance of FASTinov^®^ gramneg kit for AST directly from positive blood cultures of Gram-negative bacteria. The overall categorical agreement between FAStinov^®^ test and the ISO reference method was 98%, with a rate of minor, major and very major discrepancies of 1.03, 1.59, and 1.06%, respectively. According with Food and Drug Administration (FDA), the rate of major discrepancies attributable to a test device should be < 1.5% for individual drug comparison; the rate of major discrepancies should not exceed 3%, and the overall categorical agreement should be >90% (CDRH-Guidance, 2009). The very major discrepancies obtained with FASTinov^®^ gramneg kit were detected for amoxicillin-clavulanic acid (2.00%), gentamicin (2.78%) and meropenem (6.25%). In case of gentamicin and meropenem, the high VME rates are related with the low number of resistant strains tested (1/36 and 1/16, respectively), and represents a limitation of this study. Notably, the FASTinov^®^ gramneg kit correctly detect resistance phenotypes to amikacin, cefotaxime, ceftazidime, ciprofloxacin, colistin, and piperacillin-tazobactam, not providing false susceptible results. Regarding major discrepancies, high rates occurred for amoxicillin-clavulanic acid (3.80%) and ciprofloxacin (3.57%), exceeding the limits proposed by the FDA. Curiously, Vitek2 system also provided false resistant results for CIP, with a rate of major discrepancies of 3.57% (data not shown). In case of piperacillin-tazobactam, only one major (1.15%) and one minor (0.78%) discrepancies were observed with FASTinov^®^ gramneg kit. Some authors have reported serious rates of errors for TZP with automated systems, namely BD Phoenix, MicroScan WalkAway, Vitek, and Vitek2 (Sader et al., 2006; Juretschko et al., 2007). Simultaneously, the new Accelerate Pheno System also display high rates of VM errors for TZP (8.2%).

FASTinov^®^ gramneg kit also provided a correct detection of ESBL-producing strains (CA = 1.00). Thus, these results demonstrated the potential of FASTinov^®^ gramneg kit for detection of resistance phenotypes with a timely result within 2 h.

Due to the impossibility of a faster AST result, clinicians are frequently confronted with the need to prescribe wide spectrum antibiotics in order to minimize the risk of inappropriate therapy. However, this strategy significantly increases the selective pressure upon microorganisms and might ultimately lead to the emergence of antimicrobial resistance (Paiva, 2013). Additionally, in case of severe infections, e.g., sepsis, the time interval until the beginning of appropriate antibiotic therapy has been shown to correlate closely with outcome (Kumar et al., 2009). Kollef and Micek have suggested that, in critically ill septic patients, broad spectrum therapy should be only prescribed when considered clinically necessary, while maximizing the possibility of obtaining a valid microbiological diagnosis—de-escalation (Kollef and Micek, 2005). As soon as clinical improvement is noted and/or a culprit microorganism is identified, clinicians should then narrow the spectrum of such empiric therapy to cover only the target microorganism. However, it has been suggested that for these strategies to be effective they should be implemented as early as possible (Weiss et al., 2015). Thus, the development of laboratorial tests able to provide early information about antimicrobial susceptibility profile is urgently needed. These tests would carry the dual benefit of limiting exposure to initially inappropriate therapy and the added risks involved, while allowing for faster hence more effective de-escalation. Recently, several methodologies like PCR-based techniques, mass spectrometry, microarrays, microfluidics, cell lysis-based assays and whole-genome sequencing have all demonstrated similar ability to detect resistance in various bacterial species but with several limitations, with phenotypic assays showing superior accuracy (Pulido et al., 2013).

The FASTinov^®^ gramneg kit had the advantage of providing phenotypic and much earlier information about AST. The time-to-result of the FASTinov^®^ method was significantly shorter (2 h vs. a minimum of 24 h), potentially allowing an earlier switch to a narrow spectrum, targeted therapy, thus reducing selection pressure and minimizing exposure to inappropriate therapy and its side effects. As the most microorganisms isolated from blood cultures in our study were susceptible to the antimicrobials tested, de-escalation would be possible in about 80% of the cases. However, in the scenario of routine AST (around 48 h after the positive flag of the blood culture), with Vitek2 de-escalation was only performed in 27% of the cases.

Although the EUCAST breakpoints are species dependent, AST with FASTinov^®^ gramneg was performed without previous identification (only based on Gram stain), except in case of control strains. It was possible because the clinical isolates included in this study were very susceptible (most of strains) or very resistant to the tested drugs. However, in case of strains with MIC values closer to breakpoint concentrations FASTinov software will not inform about the phenotype before strain identification.

In summary, we found that the FASTinov^®^ kit shows excellent categorical agreement and low number of errors when compared with both the gold-standard broth-microdilution protocol and the widely used Vitek2 system. The ability of FASTinov^®^ to promote optimal use of antibiotics deserves further testing. Added benefits might arise from reduced hospital LOS, lower consumption of antibiotics and improved infection control. Large studies are needed in order to reveal the real impact of new diagnostic tools in antibiotic stewardship. Nevertheless, the FASTinov^®^ gramneg method seems to be a valuable tool to perform the AST in clinical routine.

## Author contributions

SC-d-O, CP-V, and AR contributed with the experimental design of this study. AS-D, RT-S, NM, IM-O, and AS carried out all experiments. RT-S, SC-d-O, and CP-V analyzed and interpreted the results. EP, PM, JP, and RC evaluated and discussed the potential impact of the results. SC-d-O, RT-S, and CP-V wrote the manuscript, and all authors performed a critical revision and approved the final version.

### Conflict of interest statement

AS-D, NM, IM-O, and RT-S are employees of FASTinov, S.A. SC-d-O, CP-V, and AR are co-founders of FASTinov, S.A. However, the study was designed and executed in an open manner, in Faculty of Medicine and Centro Hospitalar Sao Joao, EPE. The other authors declare that the research was conducted in the absence of any commercial or financial relationships that could be construed as a potential conflict of interest.
