# Supplementary figures and images for: Potential Impact of Flow Cytometry Antimicrobial Susceptibility Testing on the Clinical Management of Gram-Negative Bacteremia Using the FASTinov® Kit
Source: Front Microbiol. 2017 Dec 12;8:2455. doi: 10.3389/fmicb.2017.02455 (PMC5733032; doi:10.3389/fmicb.2017.02455)

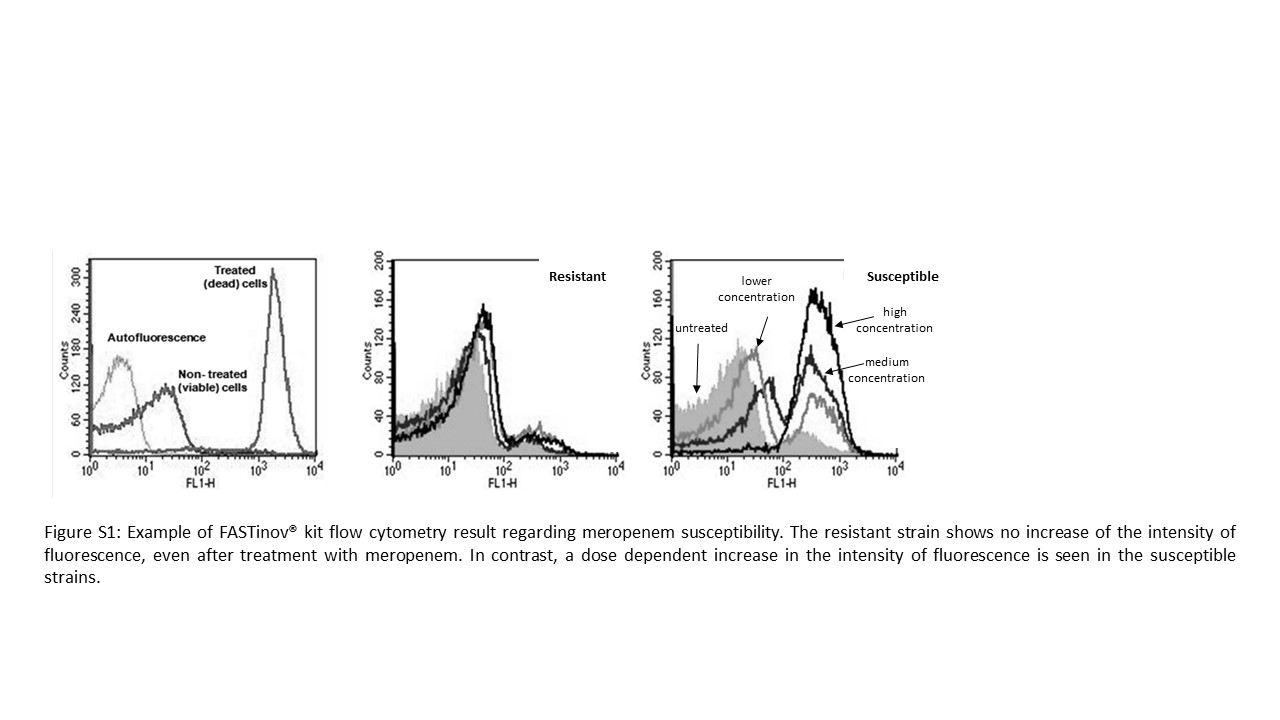

Supplement: Supplementary file 1 [file Image1.JPEG]
